# Supplementary material for: Calibration of two validated SARS-CoV-2 pseudovirus neutralization assays for COVID-19 vaccine evaluation
Source: Sci Rep. 2021 Dec 14;11:23921. doi: 10.1038/s41598-021-03154-6 (PMC8671391; doi:10.1038/s41598-021-03154-6)
Supplement: Supplementary file 1 — Supplementary Information. [file 41598_2021_3154_MOESM1_ESM.pdf]

Supplementary Material for: "Calibration of Two Validated SARS-CoV-2 Pseudovirus Neutralization Assays for COVID-19 Vaccine Evaluation"

Yunda Huang, Oleg Borisov, Jia Jin Kee, Lindsay N. Carpp, Terri Wrin, Suqin Cai, Marcella Sarzotti-Kelsoe, Charlene McDanal, Amanda Eaton, Rolando Pajon, John Hural, Christine M. Posavad, Katherine Gill, Shelly Karuna, Lawrence Corey, M. Juliana McElrath, Peter B. Gilbert, Christos J. Petropoulos, David C. Montefiori

Figure S1: Correlations of Observed (A, B) ID50 and (C, D) ID80 titers between Day 29 and Day 57 vaccinee samples measured by (A, C) Duke and (B, D) Monogram.

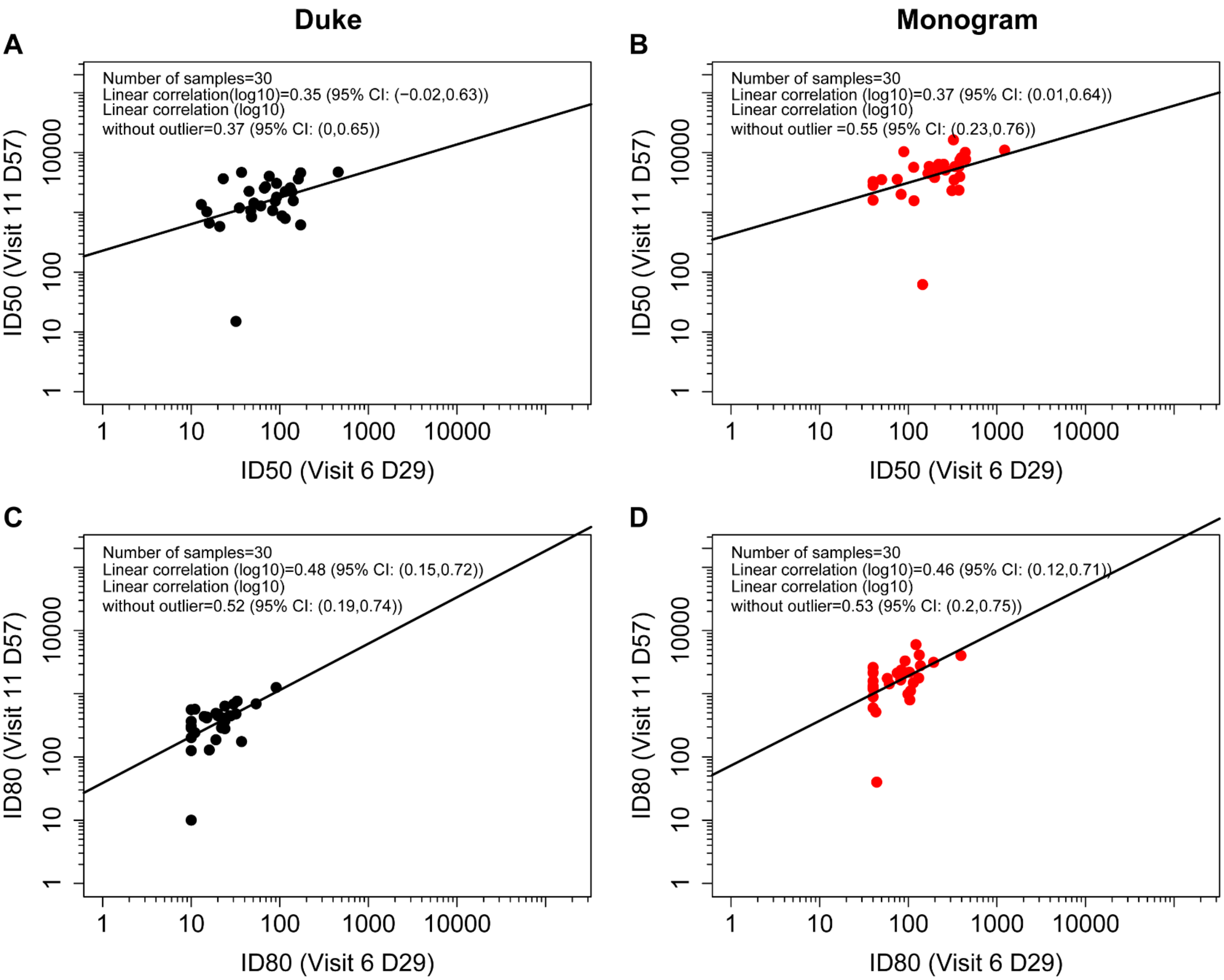

Figure S2: Comparisons of the performance of Approach 1 based on calibration factor calculated using the (A, D) arithmetic mean, (B, E) geometric mean, or (C, F) median and (A-C) ID50 or (D-F) ID80 titers. ID50, ID80 titers calibrated to the WHO International Standard, expressed in International Units per ml (IU50/ml and IU80/ml, respectively).

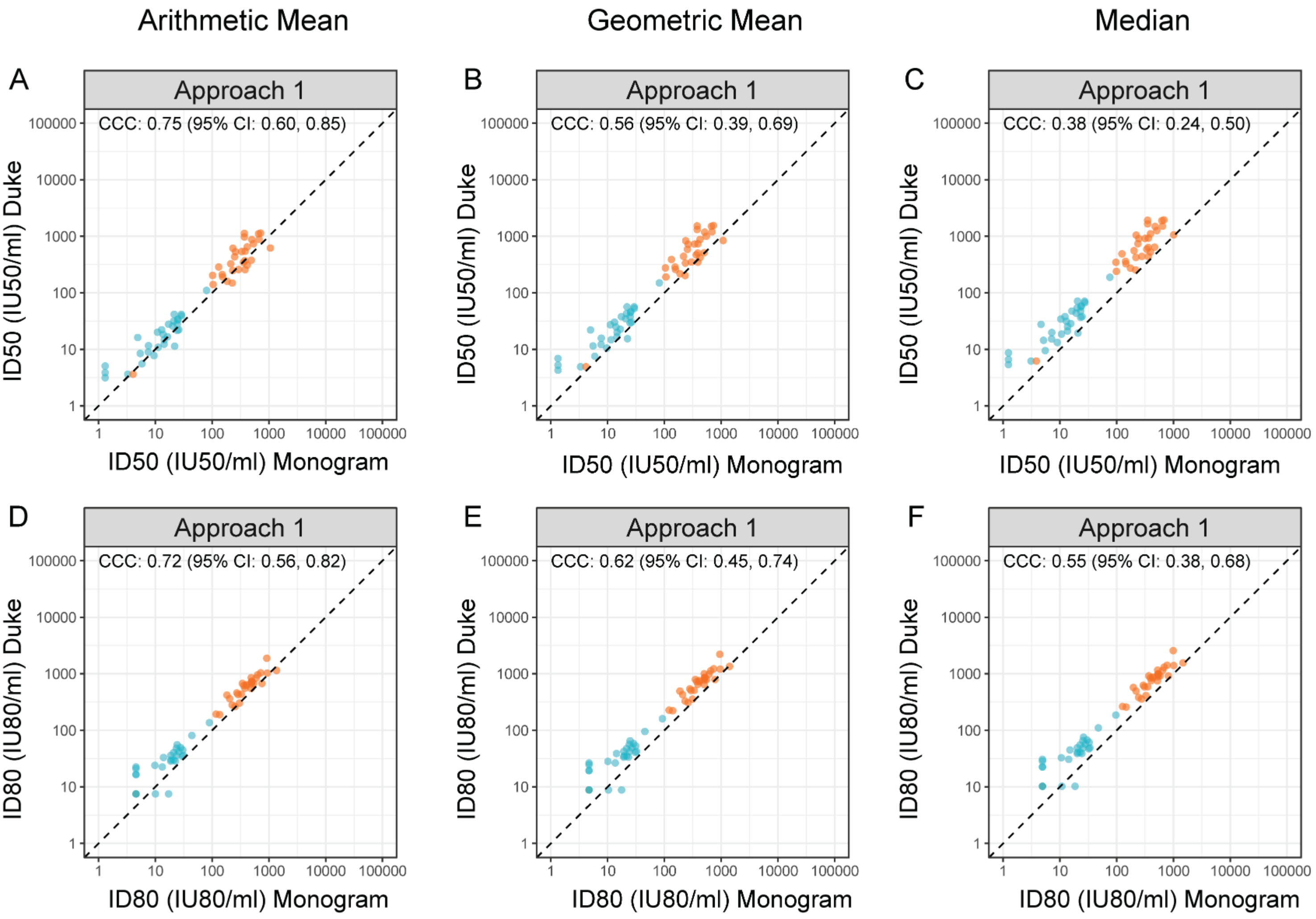

Figure S3: Scatterplots of observed (A) ID50 and (B) ID80 Duke vs. Monogram of the 248 convalescent patient samples. The solid line is the best fit linear regression line. The dashed line is the x=y diagonal line.

A

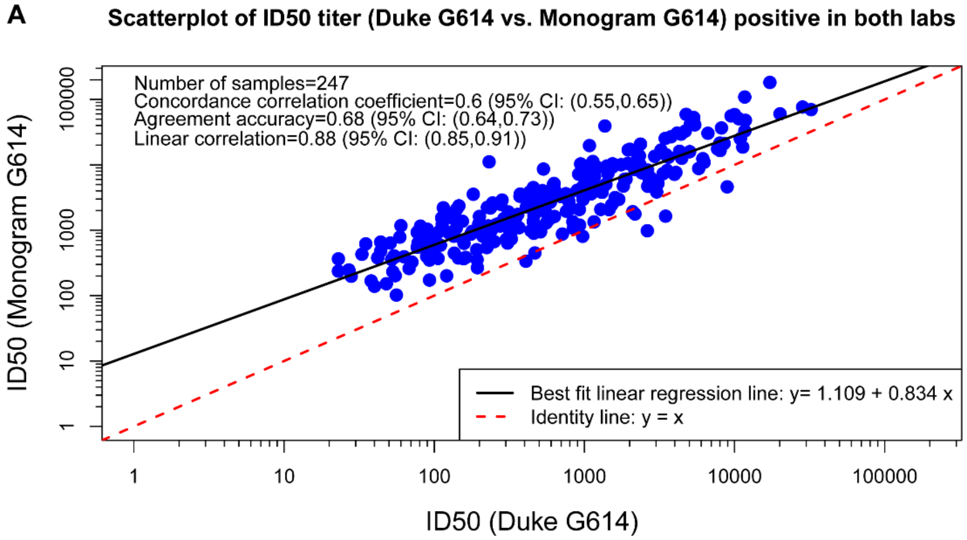

B

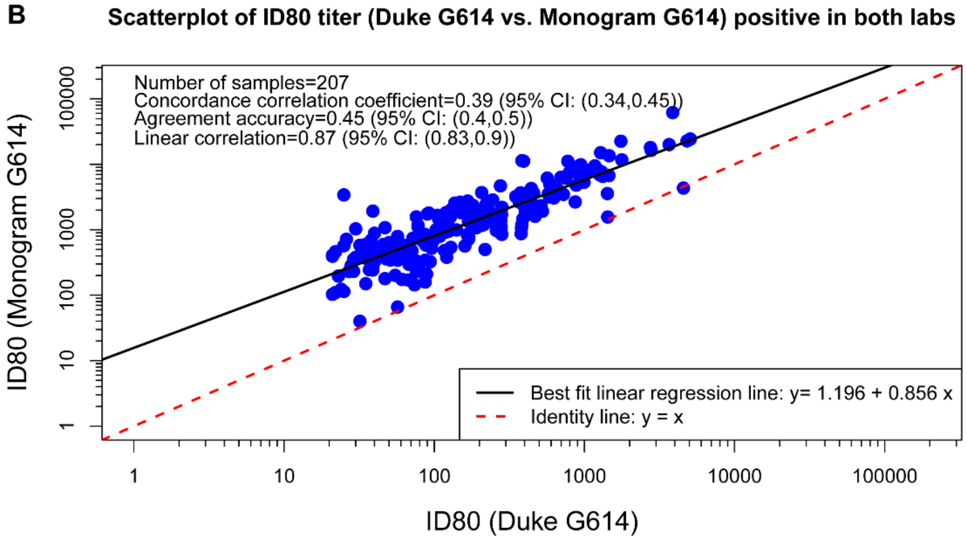

Table S1. Approach 1 Calibration Factors and Conversion Factors. The calibration factors are calculated as 1,000 (IU/mL) divided by the arithmetic mean, median or geometric mean ID50 or ID80 titers of the WHO IS sample that each lab measured on multiple vials. The conversion factors are the ratio of the calibration factors between the two labs.

Calibration	Lab	Titer	Lab-specific calibration factor	Between-lab (Duke vs. Monogram) conversion factor
Arithmetic mean	Duke	ID50	0.2418380	3.7
	Monogram		0.0652635	
	Duke	ID80	1.5010507	6.6
	Monogram		0.2281074	
Geometric mean	Duke	ID50	0.3282240	4.9
	Monogram		0.0666778	
	Duke	ID80	1.7646021	7.5
	Monogram		0.2347583	
Median	Duke	ID50	0.4129672	6.7
	Monogram		0.0619848	
	Duke	ID80	2.0449898	8.3
	Monogram		0.2461538	

Table S2. Advantages and Disadvantages of Calibration Approaches 1-3.

	Advantages	Disadvantages
**Approach 1**	• Intuitive<br>• Does not require labs running a panel of independent (convalescent) samples	• Requires running a sufficient number of vials of the WHO IS sample<br>• Does not incorporate individual-level correlations of assay readouts between labs, hence individual-level calibration may suffer from lack of accuracy.<br>• Arbitrary choice* of methods for calculating the calibration factor.
**Approach 2**	• Best performance<br>• Simple to implement when necessary paired data are available	• Requires running a panel of independent (convalescent) samples.<br>• Relies on the bivariate normal assumption and additive measurement error for assay readouts from the two labs, which may not be true.
**Approach 3**	• Intuitive	• Requires running a large panel of independent (convalescent) samples.<br>• Does not account for measurement error in the assays
